# Preoperative hypoxic biomarkers and postoperative delirium in patients with obstructive sleep apnea

**DOI:** 10.1007/s00540-024-03417-2

**Published:** 2024-11-04

**Authors:** Martin Breitkopf, Elena Ahrens, Matthias L. Herrmann, Stephanie Heinemann, Olivia Kuester, Haobo Ma, Andreas Walther, Christine Thomas, Gerhard W. Eschweiler, Christine A. F. von Arnim, Soeren Wagner

**Affiliations:** 1 Department of Anesthesiology and Intensive Care, Katharinenhospital Klinikum Stuttgart, Stuttgart, Germany; 2 Department of Anesthesia, Critical Care and Pain Medicine, Beth Israel Deaconess Medical Center, Harvard Medical School, 330 Brookline Avenue, Boston, MA 02215 USA; 3 Center for Anesthesia Research Excellence (CARE), Beth Israel Deaconess Medical Center, Harvard Medical School, Boston, MA USA; 4 Department of Neurology and Clinical Neuroscience, Faculty of Medicine and Medical Center, University of Freiburg, Freiburg, Germany; 5 Geriatric Center, Department of Psychiatry and Psychotherapy, Tübingen University Hospital, Tübingen, Germany; 6 Department of Geriatrics, University Medical Center Göttingen, Göttingen, Germany; 7 Department of Neurology, Universitaetsklinikum Ulm, Ulm, Germany; 8 Department of Old Age Psychiatry and Psychotherapy, Klinikum Stuttgart, Krankenhaus Bad Cannstatt, Stuttgart, Germany; 9 Department of Anesthesiology and Intensive Care, School of Medicine, Technical University of Munich, Klinikum Rechts Der Isar, Munich, Germany

**Keywords:** Postoperative delirium, Biomarker, OSA, Hypoxic preconditioning

## Abstract

**Purpose:**

Postoperative delirium (POD) in patients with obstructive sleep apnea (OSA) is associated with increased mortality and healthcare costs. In this study, we investigated the association of OSA risk, serum biomarkers for central nervous ischemia (S100B and NSE), and POD.

**Methods:**

After research ethics approval, patients completed the STOP BANG assessment before undergoing elective surgery. Blood was drawn for S100B and NSE measurement, and cognitive performance was tested using the Montreal Cognitive Assessment (MoCA) at study admission and postoperatively at discharge. Delirium assessment was performed using the Nursing Delirium Screening Scale (NuDESC) and the Confusion Assessment Method (CAM).

**Results:**

One hundred twenty-four enrolled patients were separated into three OSA-risk groups based on STOP BANG score testing (low risk, *n* = 22; intermediate risk, *n* = 67; high risk, *n* = 35). Preoperative NSE values increased with OSA risk (NSE in ng/ml; mean [range]; low risk: 15.6 [9.2–44.3]; intermediate risk: 21.8 [7.6–114.1]; high risk: 29.2 [10.1–151]; *p* = 0.039). Postoperative MoCA and NuDESC assessments were not different between the OSA-risk groups. We found a decreasing incidence for POD with increasing OSA risk (positive CAM: low risk: 18.1%, intermediate risk: 12.0%; high risk: 11.5%, *p* = 0.043). However, this was no longer detectable in a complete case analysis. In patients with POD, postoperative ischemic biomarker values were not different between OSA-risk groups.

**Conclusion:**

We found a trend of decreasing POD incidence with increasing OSA risk, which was not robust in a complete case analysis. Our results possibly support the phenomenon of hypoxic preconditioning.

## Introduction

Approximately 25% of patients undergoing elective surgery are affected by obstructive sleep apnea (OSA), with a prevalence in high-risk groups assumed to reach 80% [1]. In sleep apnea patients, the upper airway collapses while the respiratory drive is maintained, resulting in a cessation of respiratory gas flow and consecutively an intermittent decrease in oxygen saturation leading to hypoxia. Particularly in untreated high-risk OSA patients, dramatic events of oxygen desaturation can be observed in the body and brain, constituting a risk factor for cognitive decline [2, 3].

Postoperative delirium (POD) is a common and serious adverse event after surgery and is associated with increased mortality and a significant economic burden [4, 5]. POD is characterized by an acute onset and fluctuation of awareness and orientation [6], with an incidence ranging from 11 to 51%. [4] The overall etiology of POD is multifactorial and complex. Besides neurotransmitter imbalance, neuro-inflammation and blood barrier dysfunction [7], intraoperative hypotension and hypoxemia have recently been also described as risk factors for POD [2, 8].

In the last decade, growing evidence supports the theory of hypoxic preconditioning [9]. While hypoxic ischemic periods have suggested a cognitive decline, other authors report that, for example, slow acclimatization and duration of high-altitude exposure have a beneficial effect on cognitive function [10]. In geriatric patients, intermittent hypoxic–hyperoxic training was reported to have a beneficial effect on cognitive performance [11]. Moreover, a small observational study also found an association between high risk for OSA and lower incidence of postoperative cognitive dysfunction in patients undergoing total intravenous anesthesia for non-cardiac surgery [12]. These findings suggest that impaired cerebral oxygenation and circulation may play an important role in the development and prevention of cognitive impairment, but the role of preoperative hypoxemia is still unclear. Preconditioning phenomena have been described, suggesting that intermittent hypoxic preconditioning may play a beneficial role in postoperative neurocognitive functioning [12, 13].

Well-known serum markers of cerebral ischemia are the S100 calcium binding protein B (S100B) and the neuron-specific enolase (NSE), which can be found in low concentrations in healthy subjects. However, in patients with impaired blood brain barrier function or damage to the central nervous cells, increased serum concentrations can be detected [14]. S100B, together with glial fibrillary astrocytic protein (GFAP), is an established indicator of astrocytic activation, and is increased in cerebrospinal fluid of patients with Creutzfeld–Jacob disease, but not in Alzheimer’s disease [14].

In this prospective observational study, untreated patients with different risk of OSA were compared for the presence of elevated serum ischemia markers. We hypothesized that in untreated, suspected OSA patients, increased serum ischemia markers S100B and NSE could be detected, potentially due to recurrent, intermittent hypoxia. Moreover, due to intermittent hypoxic training like in untreated-OSA patients, we expected to detect a lower POD rate in patients at high OSA risk.

## Methods

### Study design

This study was part of a secondary analysis of data collected from 899 patients between November 2017 and April 2019 for the PAWEL study (Patient safety, cost-effectiveness and quality of life: reduction of delirium risk and postoperative cognitive dysfunction after elective procedures in older adults). The study protocol of this randomized, prospective, multicenter study was published previously [15].

For this study, baseline data from patients undergoing surgical procedures including cardiac, vascular, orthopedic and general surgery at five tertiary academic medical centers in Germany, were analyzed excluding the intervention group. Patients aged 70 years or older and scheduled for elective surgical procedures with an expected duration of at least 60 min were eligible for inclusion. Exclusion criteria were limited life expectancy of less than 15 months, insufficient German language skills and a recently diagnosed severe dementia without a health care proxy. To avoid bias, we included the baseline cohort of the PAWEL study, containing only untreated participants in terms of multimodal interventions to avoid a POD, and patients not treated for OSA.

### Data collection

Patients’ demographic and clinical data were recorded at PAWEL-study admission. These included demographic data, preoperative medical history and comorbidities, preoperative physical status as per classification of the American Society of Anesthesiologists (ASA), and risk of OSA assessed using the STOP BANG score. Moreover, perioperative data including premedication, type of surgical procedure and anesthesia, and the cut-to-suture duration time were charted. The latter data were obtained from anesthesia and surgery records.

### Risk of obstructive sleep apnea

An internationally established and validated screening tool, the STOP BANG questionnaire, was used to preoperatively detect patients at increased risk of sleep apnea [16]. The assessment focuses on questions about the occurrence of snoring, fatigue and observed nocturnal cessation of breathing, and also considers data on blood pressure, body mass index, age, neck circumference, and gender. Thus, the resulting composite score has a maximum of eight points, with a higher score-point value indicating a higher risk of OSA. Chung and co-workers published an alternative scoring model and introduced an improved STOP BANG questionnaire by specific constellations of predictive factors. Three escalation levels were described, which define a low risk at 0 to 2 points, an intermediate risk at 3 to 4 points and a high risk at 5 or more points [17]. In our study, we adopted this classification using a three-group comparison. Because, the correlation between an increasing AHI and an increasing number of risk factors in the STOP BANG screening test with regard to OSA severity has been well investigated in the literature [17]. The screening process was carried out during the PAWEL-study admission and was scheduled not later than 3 weeks before surgery.

### Ischemic biomarker blood draws

S-100B and NSE were obtained as blood-based cerebral ischemia biomarkers. Both blood biomarkers are proteins released in response to injury to central neurons and glial cells. Serum concentrations of NSE and S-100B indicate to which extent hypoxic-ischemic brain injury, for example following cardiac arrest, occurs [18]. Both markers were obtained as preoperative baseline measurements on the day of PAWEL-study admission. The second blood sample was scheduled for the 9th postoperative day. Sample preparation was performed according to standard operating procedures: After standing vertically at room temperature for 10 to 30 min the collection tubes were centrifuged at 4 °C and 2,000 g for 10 min. The serum was aliquoted and stored at -80 °C within 2 h until analysis. NSE and S100B were measured with Elecsys^®^ electrochemiluminescence immunoassays (ECLIA) for quantitative in-vitro detection of biomarkers on a cobas e411 System in Goettingen, Germany according to the manufacturers’ instructions. Lower level of detection for NSE was 0.05 ng/ml, for S100B 0.005 µg/l.

### Cognitive assessment

Cognitive function was assessed using two different neuropsychological tests. The first test was the Montreal Cognitive Assessment (MoCA) Nasredinne 2003, which was carried out as a baseline measurement on the day of PAWEL-study admission as well as on the day of discharge, but at the latest on the 10th postoperative day. The MoCA is a brief cognitive screening test for assessing cognitive impairment among older people. The test focuses on multiple cognitive domains including visuospatial ability, executive functions, memory, attention, language, abstraction, and orientation, well-established to detect patients with mild cognitive impairment and discriminate them from cognitively unhindered patients [19]. The test is available in three parallel versions to avoid learning effects.

As a second test, the Nursing Delirium Screening Scale (NuDESC) was carried out on the second and on the 6th postoperative day. The NuDESC is a five-item scale based on observations of the nurse staff assessing the functions disorientation, inappropriate behavior and communication, hallucinations, and psychomotor retardation over a 24-h period [20].

In addition, the Confusion Assessment Method (CAM) assessment was carried out on each postoperative day consecutively from the first until the eight postoperative day. With one positive-CAM assessment during any of these postoperative days, the patient was considered as delirious.

### Outcome measures

Primary outcome was the occurrence of a new cognitive impairment in terms of postoperative cognitive decline (POCD) or POD after elective surgery based on the MoCA, the NuDESC and the CAM assessment. In addition, blood-based cerebral ischemia biomarkers were obtained and correlated with OSA risk, to find potential biomarkers indicating cognitive decline in the postoperative phase.

### Statistical analyses

Low, intermediate, and high-risk patients for OSA were compared regarding the incidence of postoperative cognitive impairment. Moreover, the results were linked to blood-based cerebral ischemia biomarkers. If more than 10% of the data per patient was missing, the patient was not included in the data analysis.

To avoid bias arising from individual baseline differences in cognitive performance, we calculated the difference between first and second measurement for each patient. The individual performance was compared between groups to take into account intellectual baseline differences between patients.

Statistical analysis was performed using IBM SPSS Statistics version 27.0 for macOS platforms (IBM SPSS Inc., Chicago, IL, United States). The Chi-square test and univariate ANOVA were used to examine differences in demographic data between the three OSA-risk groups.

The univariate ANOVA was used to compare the mean scores of the three OSA-risk groups regarding biomarker levels, NuDESC, MoCA and CAM assessment. To investigate the mean differences between the individual groups, a post-hoc test was carried out using the Tukey test with variance equality. To avoid alpha error inflation due to the high number of tests, the level of significance was set at p < 0.05 for all statistical tests.

A power analysis for the ANOVA test was performed. With a medium effect size with an alpha error of 0.05 and a power of 0.85, a sample size of 122 was calculated.

## Results

### Study population and patient characteristics

**Fig. 1:**
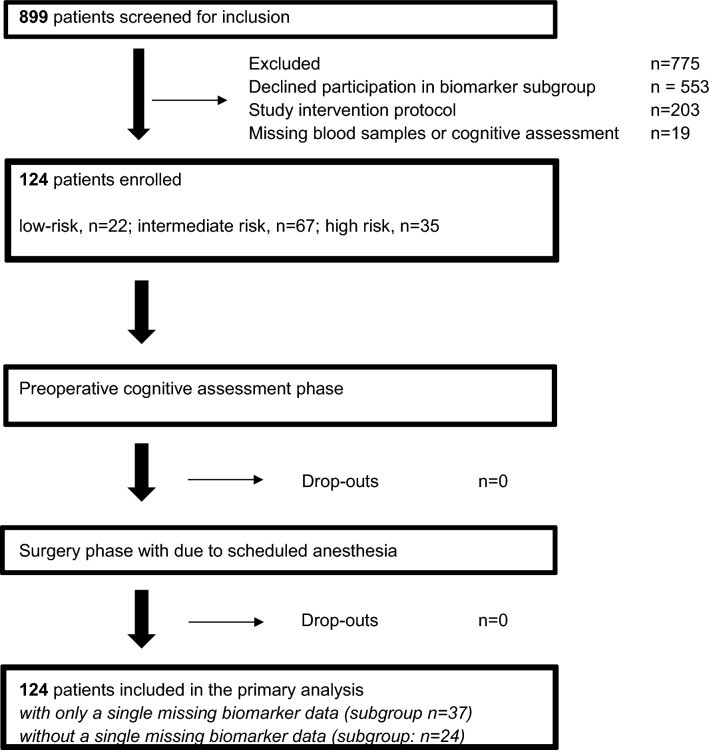
Study flow diagram

Overall, 899 patients of the baseline cohort were screened for inclusion in this study. A total of 346 patients met the inclusion criteria at the PAWEL-study admission and signed the written informed consent for this subgroup analysis before surgery. Due to PAWEL-study intervention protocol, 203 patients were excluded (Fig. 1). Notably, 553 patients declined participation in this subgroup study for personal or logistic reasons. Due to missing blood samples and incomplete or missing cognitive function records, 19 patients were excluded from further analysis. Thus, 124 patients were included at the study admission phase (Fig. 1). Among those, 24 patients had complete data regarding the second blood-harvest biomarkers in particular or only 37 patients with a single missing biomarker item.

The second phase blood samples were obtained on the 9th postoperative day on average with a broad time range (mean 9.5, standard deviation [SD] ± 5.0 days). The standard deviation is explained by an unplanned premature or delayed patient’s discharge. Based on STOP BANG score-point values, the study participants were divided into three separate groups (low risk, *n* = 22; intermediate risk, *n* = 67; high risk, *n* = 35). As per the definition of OSA risk based on the STOP BANG questionnaire, patients at higher risk for OSA were more often male, had a higher body mass index, and more often had a diagnosis of arterial hypertension (Table 1)**.**

**Table 1:** Demographic and clinical data of patients by OSA risk. Data are reported as absolute number, mean (range) or as percentage, where appropriate. OSA risk was estimated using the STOP BANG score (low risk at 0 to 2 points, intermediate risk at 3 to 4 points and high risk at 5 or more points). The chi-square test and univariate ANOVA were used to examine differences in demographic data between the three OSA risk groups. A p value < 0.05 was considered as statistically significant.

	Low risk (*n* = 22)	Intermediate risk (*n* = 67)	High risk (*n* = 35)	*p* value
Demographics				
Age (years)	77.3 (73–87)	78.7 (73–92)	79.3 (72–95)	0.282
Sex (male/female)	5/17	35/32	31/4	0.001*
Height (cm)	166.9 (155–186)	169.4 (148–191)	175.2 (152–190)	0.001*
Body weight (kg)	67.0 (45–85)	77.5 (50–145)	90.4 (70–120)	0.001*
BMI (kg/m^2^)	24.1 (15.8–32.8)	26.9 (19.7–45.8)	29.7 (21.5–43.3)	0.001*
ASA physical status	2.68 (1–4)	2.77 (2–4)	2.94 (2–4)	0.283
Anesthesia time (minutes)	175.7 (115–320)	206.3 (80–429)	191.1 (98–350)	0.295
Comorbidities				
Arterial hypertension	8 (36.4)	54 (80.6)	31 (88.6)	0.001*
Diabetes mellitus	3 (13.6)	11 (16.4)	13 (37.1)	0.551
Dementia	0 (0)	0 (0)	1 (2.9)	0.283
Alcohol abuse	0 (0)	1 (1.5)	1 (2.9)	0.707
Nicotine abuse (> 5 per day)	1 (4.5)	2 (3.0)	3 (8.6)	0.567
Heart failure	1 (4.5)	9 (13.4)	4 (11.4)	0.527

*OSA*: obstructive sleep apnea, *BMI*: body mass index.

### Primary analysis

In an unadjusted analysis including the entire cohort of 124 patients, we found significantly higher preoperative NSE values in patients at high risk for OSA (Table 2). There were no differences between the groups in preoperative S100B values and MoCA performance. Analyses of postoperative-CAM assessments demonstrated that POD occurs less frequently between the low-risk OSA to high-risk OSA patients (positive CAM: low risk: 18.1%, intermediate risk: 12.0%; high risk: 11.5%, *p* = 0.043). Low (STOP BANG ≤ 2) compared to intermediate and high (STOP BANG ≥ 3) OSA risk was not associated with a lower risk of POD in unadjusted logistic regression analysis (OR 0.91, 95% CI 0.64–1.34, *p* = 0.14). We found no differences between the three OSA-risk groups in levels of NSE or S100B, as well as NuDESC or MoCA assessment results in the postoperative phase (Table 2). Moreover, by analyzing the values of pre-post changes no difference between low, intermediate, and high-risk OSA patients was found in changes of NSE and S100B levels or changes in NuDESC and MoCA scores (Table 2).

**Table 2:** Primary analysis. Results of the pre- and postoperative cognitive testing and results of hypoxic biomarker between each group. Data are reported as median (range). The p values derived from ANOVA. *: significant differences with *p* < 0.05. OSA risk was estimated using the STOP BANG score (low risk at 0 to 2 points, intermediate risk at 3 to 4 points and high risk at 5 or more points)

	Preoperative values				Postoperative values				Difference			
Complete cohort *n* = 124	Low risk (*n* = 22)	Intermediate risk (*n* = 67)	High risk (*n* = 35)	*p* value	Low risk (*n* = 22)	Intermediate risk (*n* = 67)	High risk (*n* = 35)	*p* value	Low risk (*n* = 22)	Intermediate risk (*n* = 67)	High risk (*n* = 35)	*p* value
NSE ng/ml	15.6 (9.2–44.3)	21.8 (7.6–114.1)	29.2 (10.1–151)	0.039*	30.3 (13.5–55.3)	41.4 (12.7–144.5)	61.3 (13.5–320.9)	0.395	13.2 (-12.9–43.5)	19.6 (-12.7–136.8)	22.1 (-12.1–169.8)	0.869
S100B µg/l	0.160 (0.03–0.27)	0.157 (0.03–0.59)	0.143 (0.02–0.43)	0.723	0.152 (0.08–0.23)	0.177 (0.05–0.37)	0.186 (0.07–0.31)	0.551	0.02 (-0.06–0.03)	-0.002 (-0.43–0.30)	-0.005 (-0.05–0.10)	0.771
MoCA	23.7 (17–29)	23.8 (14–29)	22.9 (10–29)	0.531	23.4 (18–30)	23.3 (14–30)	22.2 (12–29)	0.425	-0.6 (-8–4)	-0.7 (-7–6)	-0.9 (-6–4)	0.920
	2nd postoperative day				6th postoperative day				Difference			
NuDESC	0.59 (0–8)	0.29 (0–7)	0.67 (0–6)	0.385	0.6 (0–6)	0.11 (0–1)	0.29 (0–5)	0.336	0.5 (0–2)	0.3 (-1–7)	0.6 (0–4)	0.695

*NSE*: Neuron-Specific Enolase, *S100B*: S100 calcium binding protein B, *MoCA*: Montreal Cognitive Assessment, *NuDESC*: Nursing Delirium Screening Scale

### Sensitivity and exploratory analyses

To avoid bias arising from missing data values in the primary analysis, we repeated the analysis in cases with only one missing data parameter (*n* = 37, Table 3) and with a data set without missing parameters (*n* = 24, Table 4). In these analyses, we did not find significant differences between the three OSA-risk groups and all studied outcomes.

**Table 3:** Subgroup analysis with one missing value. Results of the pre- and postoperative cognitive testing and results of hypoxic biomarker between each group. Data are reported as median (range). The p values derived from ANOVA. *: significant differences with p < 0.05. OSA risk was estimated using the STOP BANG score (low risk at 0 to 2 points, intermediate risk at 3 to 4 points and high risk at 5 or more points)

	Preoperative values				Preoperative values				Difference			
Subgroup with one missing *n* = 37	Low risk (*n* = 9)	Intermediate risk (*n* = 20)	High risk (*n* = 8)	*p* value	Low risk (*n* = 9)	Intermediate risk (*n* = 20)	High risk (*n* = 8)	*p* value	Low risk (*n* = 9)	Intermediate risk (*n* = 20)	High risk (*n* = 8)	*p* value
NSE ng/ml	17.1 (9.2–44.4)	22.1 (7.6–49.7)	39.9 (10.1–151.0)	0.126	30.3 (13.5- 55.3)	42.4 (12.7–144.5)	64.6 (16.6–320.9)	0.442	13.2 (-12.9–43.5)	20.3 (-12.7–136.8)	24.7 (-12.1–169.8)	0.820
S100B µg/l	0.18 (0.05–0.27)	0.16 (0.03–0.59)	0.20 (0.13–0.23)	0.572	0.15 (0.08–0.23)	0.17 (0.05–0.38)	0.20 (0.07–0.31)	0.362	0.02 (-0.07–0.03)	-0.01 (-0.43–0.30)	-0.01 (-0.06–0.10)	0.742
MoCA	24 (17–29)	23.1 (17–28)	24.0 (16–29)	0.788	23.6 (19–29)	23.0 (14–30)	21.8 (14–27)	0.642	-0.2 (-4–2)	-0.12 (-4–5)	-2.25 (-6–3)	0.097
	2nd postoperative day				6th postoperative day				Difference			
NuDESC	1.1 (0–8)	0.4 (0–6)	0.25 (0–2)	0.497	1.2 (0–6)	0.2 (0–1)	0 (0)	0.200	0.8 (0–2)	0.33 (-1–6)	0.33 (0–2)	0.794

*NSE*: Neuron-Specific Enolase, *S100B*: S100 calcium binding protein B, *MoCA*: Montreal Cognitive Assessment, *NuDESC*: Nursing Delirium Screening Scale

**Table 4:** Subgroup analysis without missing values Results of the pre- and postoperative cognitive testing and results of hypoxic biomarker between each group. Data are reported as median (range). The p values derived from ANOVA. *: significant differences with *p* < 0.05. OSA risk was estimated using the STOP BANG score (low risk at 0 to 2 points, intermediate risk at 3 to 4 points and high risk at 5 or more points)

Test	Preoperative values				Postoperative values				Difference			
Subgroup without missing n = 24	Low risk (*n* = 5)	Intermediate risk (*n* = 13)	High risk (*n* = 6)	*p* value	Low risk (*n* = 5)	Intermediate risk (*n* = 13)	High risk (*n* = 6)	*p* value	Low risk (*n* = 5)	Intermediate risk (*n* = 13)	High risk (*n* = 6)	*p* value
NSE ng/ml	21.4 (9.2–44.4)	23.3 (10.7–49.7)	22.0 (10.1–42.7)	0.961	31.4 (13.5– 55.3)	40.1 (13.1–142.7)	28.6 (16.6–47.9)	0.657	10.0 (−12.9–24.5)	16.7 (−2.7–93.0)	6.6 (5.0–8.2)	0.571
S100B µg/l	0.16 (0.05–0.27)	0.19 (0.03–0.59)	0.20 (0.13–0.23)	0.843	0.14 (0.08–0.23)	0.18 (0.06–0.25)	0.19 (0.07–0.31)	0.425	0.02 (-0.07–0.03)	−0.01 (−0.43–0.07)	−0.01 (−0.06–0.10)	0.975
MoCA	24 (17–27)	22.8 (17–27)	23.2 (16–29)	0.823	22.6 (19–26)	22.6 (14–30)	20.8 (14–26)	0.695	−1.4 (−4–2)	−0.15 (-4–3)	−2.33 (−6–3)	0.257
	2nd postoperative day				6th postoperative day				Difference			
NuDESC	2.0 (0–8)	0.62 (0–6)	0.33 (0–2)	0.348	1.2 (0–6)	0.15 (0–1)	0 (0)	0.213	0.8 (0–2)	0.46 (0–6)	0.33 (0–2)	0.852

*NSE*: Neuron-Specific Enolase, *S100B*: S100 calcium binding protein B, *MoCA*: Montreal Cognitive Assessment, *NuDESC*: Nursing Delirium Screening Scale

With an exploratory intent, we investigated the differences in mean preoperative NSE values between the groups and found significant differences between the low and the high OSA risk group (Table 5).

**Table 5:** Entire group analysis, presented are the results of post-hoc analysis of the significant results from Table 2. Data are reported as differences in mean. The p value is the significance level of the difference between the single three OSA risk groups as a post-hoc analysis within the ANOVA analysis from Table 2; *: significant differences with *p* < 0.05*.* OSA risk was estimated using the STOP BANG score (low risk at 0 to 2 points, intermediate risk at 3 to 4 points and high risk at 5 or more points)

Post-hoc test tukey			
Complete cohort *n* = 124	Low risk Difference mean	Intermediate risk Difference mean	High risk Difference mean
NSE ng/ml preoperative			
Low risk (*n* = 22)		−6.1 (*p* = 0.404)	−13.6 (*p* = 0.034*)
Intermediate risk (*n* = 67)	6.1 (*p* = 0.404)		−7.4 (0.180)
High risk (*n* = 35)	13.6 (*p* = 0.034*)	7.4 (*p* = 0.180)	

*NSE*: Neuron-Specific Enolase, *S100B*: S100 calcium binding protein B, *MoCA*: Montreal Cognitive Assessment, *NuDESC*: Nursing Delirium Screening Scale

### Postoperative biomarkers

To quantify whether the biomarkers used in OSA patients with delirium change significantly and thus serve as biomarkers for a POD, we compared postoperative biomarker values in patients with delirium based on OSA risk. There was no association between OSA risk and biomarker levels (Table 6).

**Table 6:** Subgroup analysis of patients with NuDESC score ≥ 2 or MoCA score < 23 and positive CAM assessment, respectively. Data are reported as median (range). The p value is the significance level of the difference between the two OSA risk groups; *: significant differences with p < 0.05. OSA risk was estimated using the STOP BANG score (low risk at 0 to 2 points, intermediate risk at 3 to 4 points and high risk at 5 or more points)

Subgroup with delirium based on NuDESC ≥ 2 or MoCA < 23			
	Low risk (*n* = 13)	Intermediate-to-high risk (*n* = 42)	*p*-value
NSE ng/ml	36.3 (20.0–55.3)	43.5 (15.3–142.7)	0.625
S100B µg/l	0.15 (0.08–0.23)	0.20 (0.11–0.31)	0.061
Subgroup with delirium based on positive CAM assessment			
	Low risk (*n* = 4)	Intermediate-to-high risk (*n* = 12)	*p* value
NSE ng/ml	32.3 (21.3–49.5)	40.1 (16.7–101.3)	0.253
S100B µg/l	0.16 (0.09–0.21)	0.19 (0.12–0.31)	0.125

*NSE*: Neuron-Specific Enolase, *S100B*: S100 calcium binding protein B, *MoCA*: Montreal Cognitive Assessment, *NuDESC*: Nursing Delirium Screening Scale

## Discussion

In this subgroup analysis of the prospective PAWEL study, we found higher preoperative NSE values in patients at high risk for OSA. While there were no differences between the three OSA-risk groups in levels of NSE or S100B, as well as NuDESC or MoCA assessment results in the postoperative phase, we detected a decreasing CAM-based POD risk with increasing OSA risk. However, our results did not remain robust in a complete case analysis and our data do not support the hypothesis of cerebral ischemic biomarker-based risk detection regarding POD development in OSA-suspect patients.

The biomarker S100B expressed in astrocytes reflects the blood brain barrier integrity and permeability. This blood brain barrier can be disturbed by inflammatory processes as they are also described in OSA, therefore elevated plasma levels of S100B were hypothesized [3, 21]. NSE is a biomarker for hypoxic brain damage, which is also linked in OSA patients and mediated by the recurring, intermittent nocturnal oxygen depletion and consecutive hypoxia [22]. Elevated S100B plasma levels but not NSE levels have been reported for individuals affected by an obstructive sleep apnea syndrome [22, 23]. However, others found both ischemic biomarkers to be elevated [24–26]. Moreover, it was reported that during continuous positive airway pressure (CPAP) treatment, both elevated ischemic biomarkers decreased and cognitive performance measured by MoCA assessment improved after 3 months. [25] We found no significant differences in a complete case analysis in S100B or NSE-plasma concentrations. This might be explained by the fact that our study patients were screened using the STOP BANG questionnaire. Thus, we included highly susceptible OSA patients while previous studies reported findings in diagnosed OSA patients.

Our findings are supported by a study of Jordan and co-workers, who did not detect any differences in S100B and NSE level in OSA patients and assumed that the structural cerebral brain injury might be too small to produce a detectable serum level increase [27]. Braga and colleagues reported in a small sample of 29 polysomnography-based diagnosed OSA patients compared to 17 male asymptomatic patients, that NSE levels were similar in both groups, while S100B levels were elevated in the OSA group [22]. Thus, studies focusing on cerebral ischemic biomarkers remain inconsistent and conflicting, due to different blood-collection schedules and diagnostic criteria for OSA, even if all authors unanimously suspected cerebral hypoxic cell damage as the underlying mechanism. However, we also unfortunately failed to demonstrate any statistical difference in ischemic biomarker analysis.

Neurocognitive impairment as a result of intermittent hypoxia is assumed in suspected OSA patients and is also considered as a risk factor for POD [8]. Several studies reported an association between OSA and POD risk [28–30]. While we found no significant difference between the OSA-risk groups focusing on postoperative MoCa, or postoperative NuDESC assessment, an observational study including 66 patients reported an association between high STOP BANG scores and a cognitive decline 6 weeks after surgery [31]. In contrast, an observational study including more than 1,400 patients and the same STOP BANG grouping as our study found in an unadjusted analysis a significant association between high risk of OSA and POD. However, after thorough adjustment, the association was statistically insignificant, which is in line with our findings [32].

We found in our data analysis that with an increasing risk for OSA, the risk for POD decreased. Positive effects of hypoxic preconditioning on cognitive function were already described in the literature several years ago [33]. Initially, positive results were found in animal studies [34]. Hoth and co-workers subsequently found in a study of patients with newly diagnosed OSA that more severe hypoxemic events were associated with an improvement in memory [35]. A recently published randomized controlled trial including 40 young male participants treated with remote ischemic preconditioning for 7 days found a significantly improved spatial memory function during acute high-altitude exposure as an indicator for cognitive function [36]. In an observatory study with untreated high-risk OSA patients postoperative cognitive disorder was significantly lower than in a control group treated by total intravenous anesthesia [12]. However, in a similarly designed study using a volatile anesthesia approach, the postoperative cognitive impairment in the control group was attenuated [13]. Nevertheless, the authors likewise assumed preconditioning effects in untreated-OSA patients.

### Limitations

There are limitations to our study that need to be discussed. First, the obtained cerebral ischemic biomarker S100B was measured from peripheral blood samples, which might not accurately represent the corresponding values in the brain circulation. Usually, the S100B value in peripheral blood is lower than in cerebrospinal fluid samples [37], which might have led to an underestimation. Furthermore, we harvested the blood samples at a different time than others, which leads to difficulties when aiming for an exact comparison with previous studies [38]. In our study, no standardized collection times could be defined because the blood-sample analysis of the specific biomarkers was not available on every day. Second, delirium assessment using a single test might not be sufficient. However, the CAM assessment is a well-established instrument to detect patients affected by delirium. Due to the differences in the delirium test systems and the fact that subjective influence of the raters themselves cannot be excluded finally, the statistical evaluation may have been influenced, so that a positive effect is detected by using the CAM Test, but no rating with the MoCa and NuDesc. Likewise, OSA screening using the STOP BANG score might have limited sensitivity. However, several studies have demonstrated the STOP BANG score’s high efficiency and accuracy in OSA screening [39]. However, we grouped OSA patients and calculated the POD association to different severity OSA levels as previously published [17]. Finally, our results did not remain robust in a complete case analysis, thus only show a small trend, and we included only a small number of patients from a single institution, resulting in limited generalizability.

## Conclusion

In this study, high risk for OSA was associated with elevated preoperative NSE values and a decreasing CAM-based POD risk with increasing OSA risk. However, our results did not remain robust in a complete case analysis and our data do not support the hypothesis of risk detection for POD using blood-stream-based ischemic biomarkers. Future studies should investigate to what extent hypoxic preconditioning can have a positive effect on postoperative cognitive impairment in OSA patients.

## Data Availability

The datasets generated and analyzed during the current study are available from the corresponding author on reasonable request.
